# Cooking up a nutritious curriculum

**DOI:** 10.1080/10872981.2019.1679951

**Published:** 2019-10-22

**Authors:** Gayle Tin Yee Fung, Jonathan Seng-Sung Mak

**Affiliations:** Barts and the London School of Medicine and Dentistry, Queen Mary University of London, London, UK

Dear Editor,

We have recently had the pleasure of reading the article by Pang et al. [1] detailing their implementation of a unique preclinical culinary and nutrition course. As a final year medical students in the UK, we would like to thank the authors for their intriguing paper and make some contributions. We agree that addressing gaps in knowledge surrounding nutrition and patient education is a vital part of preventative medicine, especially with obesity and other lifestyle-related diseases on the increase. We also agree that nutrition education is lacking not only in the US, but in Europe as well, where students feel nutrition education is deficient [2].

A study by Mogre et al. [3] has shown that students not only feel a lack of nutrition education, but also the lack of faculty to enable this and poor collaboration with nutrition professionals. The paper by Pang et al. [1] has elegantly designed a course addressing these areas. One of their main aims was to improve the ability of a future doctor to counsel their patient on diet and nutrition and, ultimately, make a clinical impact. This was mentioned in the text but not reflected in the course syllabus provided. There is also a little reference to which diseases were focused on, it would be interesting to see what type of meal preparation was taught for each disease and the evidence behind the recommendations.

They also succeeded in improving the students’ ability to cook healthy meals for themselves where large effect sizes were achieved. This effectively combats the issue of imposter syndrome and empowers them with the confidence to give advice. However, this does not objectively demonstrate improved student ability to counsel patients, which is the most clinically significant endpoint. The pre and post-course surveys use surrogate markers for this, e.g. subjective confidence, food identification skills, knowledge in culinary technique. We suggest a cohort study where one group participates in the course and the other does not with objective testing at the end in the form of an OSCE. This could provide more evidence and power to support the case for increased incorporation of nutrition into the medical curriculum. This has previously been shown to be effective by Taren et al. [4] who demonstrated significant improvement in mean OSCE scores after implementation of their nutrition curriculum.

This also leads us to wonder which skills from the culinary course are necessary and whether the syllabus could be streamlined to be more efficient. For example, it may not be a doctor’s duty to create budgeted meal plans for the patient, but rather know which resources to signpost a patient towards, i.e. a dietician. Furthermore, aside from learning how to counsel patients to make positive dietary changes, we could also help trainees learn how to do so in a time-pressured environment as many patient encounters are fleeting.

To conclude, this study has highlighted again the lack of nutrition education in medical schools and demonstrate some of the beneficial effects of a well-designed culinary course on the training student. However, there needs to be work done to translate these benefits in a clinical environment and show this in an objective manner so that educational bodies will have more motivation and justification in dedicating more attention to nutrition education within medical training.
